# Porcine Adipose-Derived Mesenchymal Stem Cells Retain Their Stem Cell Characteristics and Cell Activities While Enhancing the Expression of Liver-Specific Genes after Acute Liver Failure

**DOI:** 10.3390/ijms17010062

**Published:** 2016-01-05

**Authors:** Chenxia Hu, Ning Zhou, Jianzhou Li, Ding Shi, Hongcui Cao, Jun Li, Lanjuan Li

**Affiliations:** Collaborative Innovation Center for Diagnosis and Treatment of Infectious Diseases, State Key Laboratory for Diagnosis and Treatment of Infectious Diseases, School of Medicine, First Affiliated Hospital, Zhejiang University, Hangzhou 310006, China; 11318093@zju.edu.cn (C.H.); hxgczn@163.com (N.Z.); lijianzhou1986@163.com (Ji.L.); shiding@zju.edu.cn (D.S.); hccao@zju.edu.cn (H.C.); lijun2009@zju.edu.cn (Ju.L.)

**Keywords:** acute liver failure (ALF), adipose-derived mesenchymal stem cells (ADMSCs), biological characteristics, autotransplantation

## Abstract

Acute liver failure (ALF) is a kind of complicated syndrome. Furthermore, adipose-derived mesenchymal stem cells (ADMSCs) can serve as a useful cell resource for autotransplantation due to their abundance and micro-invasive accessability. However, it is unknown how ALF will influence the characteristics of ADMSCs and whether ADMSCs from patients suffering from end-stage liver diseases are potential candidates for autotransplantation. This study was designed to compare various properties of ALF-derived ADMSCs with normal ADMSCs in pig models, with regard to their cellular morphology, cell proliferative ability, cell apoptosis, expression of surface antigens, mitochondrial and lysosomal activities, multilineage potency, and expression of liver-specific genes. Our results showed that ALF does not influence the stem cell characteristics and cell activities of ADMSCs. Intriguingly, the expression levels of several liver-specific genes in ALF-derived ADMSCs are higher than in normal ADMSCs. In conclusion, our findings indicate that the stem cell characteristics and cell activities of ADMSCs were not altered by ALF and these cells can serve as a new source for regenerative medicine.

## 1. Introduction

The liver is one of the few organs with an immense regeneration potential. At one time, the unique suitable therapy available for patients suffering from advanced or irreversible liver diseases was liver transplantation [1]. Because liver transplantation is limited by the high medical expenses, the scarcity of donors and unavoidable immunosuppression, mesenchymal stem cell (MSC) therapy provides a promising alternative to liver transplantation [2]. Bone marrow mesenchymal stem cells (BMMSCs), which are MSCs that reside within the bone marrow niche, possess the advantages of convenient collection, rapid proliferation, a persistent self-renewal capacity, and a lack of ethical issues [3]. However, traditional bone marrow procurement is distressful for patients and yields low numbers of MSCs, which has led to the application of alternative MSC sources, outside of the bone marrow niche. The quantity of MSCs derived from adipose tissue is extremely bigger than that derived from bone marrow [4]. In addition, the extraction of adipose tissue causes minimal patient discomfort compared with the extraction of bone marrow. Adipose-derived mesenchymal stem cells (ADMSCs) demonstrated the longest culture period and the optimal proliferative ability compared with MSCs derived from other tissues [5]. Thus, adipose tissue is likely to be an ideal source for less invasive procedures and may provide larger amounts of autologous stem cells than other types of MSCs. Regarding liver disease therapy, these cells can be used to treat hepatectomy [6], liver cirrhosis [7], liver failure [8], fulminant hepatitis [9] and other lethal liver diseases. Multiple clinical trials are currently registered worldwide, additionally liver and pulmonary fibrosis are most widely represented for evaluating MSC therapy [10].

Acute liver failure (ALF) is a kind of complicated syndrome that induces sequential changes and rapid alterations of cytokines *in vivo*. Liver injury precisely activates progenitor cells in liver tissue, and the resulting cells can proliferate and differentiate into mature hepatocytes *in vivo* [11]. Serum which was isolated from liver injured rats enhances the hepatic differentiation efficacy of BMMSCs [12] and is more efficient than hepatocyte growth factor (HGF), suggesting that certain pathological environments may influence the characteristics of MSCs. However, it is unknown how ALF will influence ADMSCs *in vivo* and whether ADMSCs isolated from patients suffering from end-stage liver diseases are potential candidates for autotransplantation. Pigs can serve as an excellent model for studying new therapies for various diseases according to their similar anatomy and physiology to humans [13]. Preclinical studies have demonstrated that these cells were able to mediate their therapeutic effects by hepatic differentiation, paracrine stimulation and neovascularization of regenerating liver [14].

In the present study, we set out to acquire ALF pig models and analyze various properties of ALF-derived ADMSCs, including their cellular morphology, cell proliferation, cell apoptosis, surface antigen expression, mitochondrial and lysosomal activities, adipogenic and osteogenic differentiation, and liver-specific gene expression. The findings help us to clarify whether ADMSCs from patients with end-stage liver diseases can serve as a candidate source for cell autotransplantation.

## 2. Results

### 2.1. Cell Morphology

**Figure 1:**
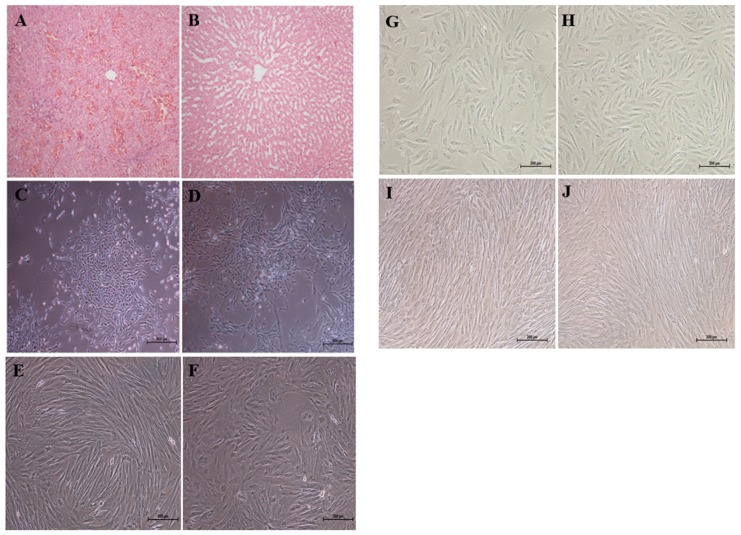
Liver tissue specimens were collected for histochemistry and immunohistochemistry to confirm the acute liver failure (ALF) group and normal group. Morphology of ALF-derived adipose-derived mesenchymal stem cells (ADMSCs) and normal ADMSCs under a light microscope. (**A**) Extensive hepatocyte necrosis and hemorrhaging and a collapsed hepatic lobular structure were observed in the post-mortem liver tissue specimen of the ALF group; (**B**) The normal liver tissue showed normal liver lobule structures; (**C**) Morphology of ALF-derived ADMSCs at day 15; (**D**) Morphology of normal ADMSCs at day 15; (**E**) Morphology of ALF-derived ADMSCs at passage 5; (**F**) Morphology of normal ADMSCs at passage 5; (**G**) Morphology of ALF-derived ADMSCs at passage 10; (**H**) Morphology of normal ADMSCs at passage 10; (**I**) Morphology of ALF-derived ADMSCs at passage 15; (**J**) Morphology of normal ADMSCs at passage 15; (**A**,**B**) (Hematoxylin and eosin (HE), ×10); (**C**,**D**) Scale bars = 500 μm; (**E**–**J**) Scale bars = 200 μm; FADMSCs (ADMSCs derived from ALF pigs) are ADMSCs derived from ALF pigs, and NADMSCs (ADMSCs derived from normal pigs) are ADMSCs derived from normal pigs in all figures.

ALF was confirmed by liver histopathology. Extensive hepatocyte necrosis and hemorrhaging and a collapsed hepatic lobular structure were observed in the post-mortem liver tissue specimen of the ALF group. (Figure 1A), and the normal liver tissue showed normal liver lobule structures (Figure 1B). Our morphological observations showed that ALF-derived ADMSCs (Figure 1C) and normal ADMSCs (Figure 1D) proliferated slowly and formed colonies of densely packed small cells through 15 days of culture, after which parallel or vortex-like patterns, abundant cytoplasm and large nuclei were observed. After 20 days of culture, adherent cells were passaged. These cells then proliferated quickly to confluence after culturing for 5–6 days. The morphology of both the ALF-derived ADMSCs (Figure 1E) and normal ADMSCs (Figure 1F) at passage 5 demonstrated parallel or vortex-like patterns. The morphology of both the ALF-derived ADMSCs (Figure 1G) and normal ADMSCs (Figure 1H) changed the cytoplasmic volume and content as well as an increase of perinuclear granularity after passage 10, while at passage 15, a round or flat shape of both cell types was observed under a light microscope (Figure 1I–J).

### 2.2. Cell Proliferative Capacity

**Figure 2:**
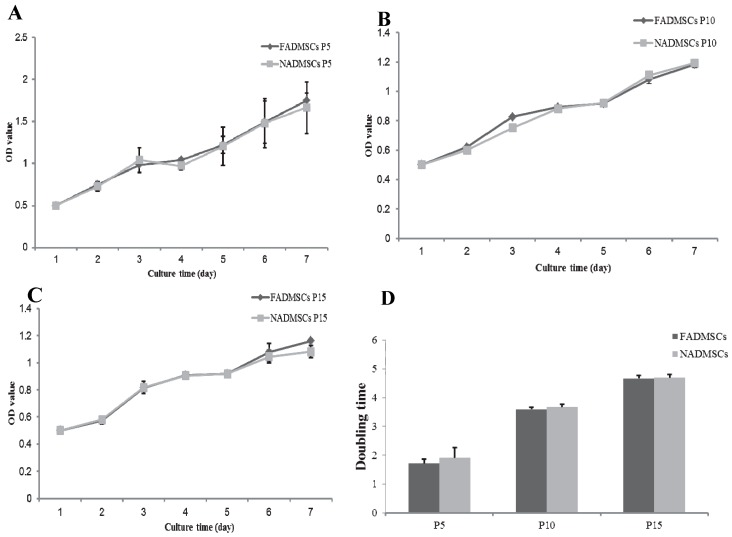
Comparison of the proliferative capacity between ALF-derived ADMSCs and normal ADMSCs. Measurements were performed based on water soluble tetrazolium salt (WST)-1 assays. The optical density (OD) value indicates the optical density at 450 nm. (**A**) Comparative analysis of cell proliferation between the two groups at passage 5; (**B**) Comparative analysis of cell proliferation between the two groups at passage 10; (**C**) Comparative analysis of cell proliferation between the two groups at passage 15; (**D**) Comparative analysis of the doubling time between the two groups at passages 5, 10 and 15. The results are representative of three independent experiments. Mean ± SD values are plotted.

We found that the proliferation rates of both groups of cells at passage 5 (Figure 2A), passage 10 (Figure 2B), and passage 15 (Figure 2C) were very slow during the first 2–3 days, accelerated at a growing rate during 4–6 days, and then slowed thereafter. The doubling time of ALF-derived ADMSCs at passage 5 in logarithmic phase was 1.71 ± 0.15 days, while the doubling time of normal ADMSCs at passage 5 was 1.90 ± 0.36 days, and there was no significant difference between ALF-derived ADMSCs and normal ADMSCs (*p* > 0.05) (Figure 2D). When these cells were expanding to passage 10, the doubling time of ALF-derived ADMSCs was 3.59 ± 0.08 days, and the doubling time of normal ADMSCs was 3.67 ± 0.09 days. At passage 15, the doubling time of ALF-derived ADMSCs was 4.66 ± 0.12 days, and the doubling time of normal ADMSCs was 4.69 ± 0.12 days. There was no significant difference between the two groups even when they were passaged several times. Our results demonstrated that the proliferative ability of ADMSCs is not altered after ALF, and the proliferative ability of ALF-derived ADMSCs were comparable to normal ADMSCs at late passages.

### 2.3. Cell Apoptosis

**Figure 3:**
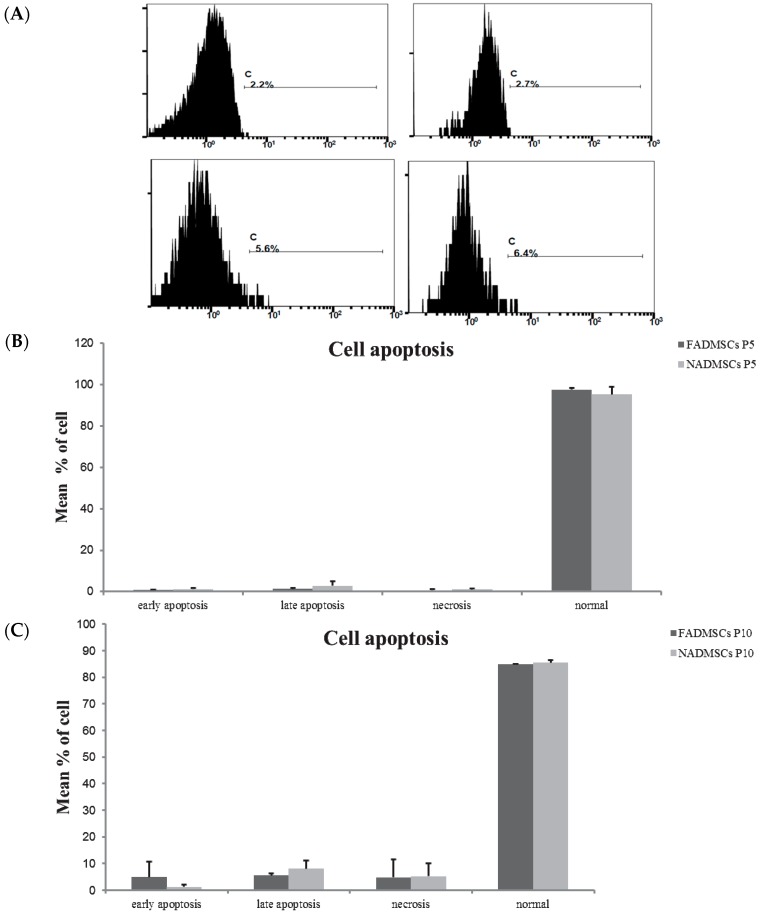
Apoptosis rates in ALF-derived ADMSCs and normal ADMSCs determined via flow cytometry. (**A**) Examples of apoptosis rate in ALF-derived ADMSCs and normal ADMSCs; (**B**) Early apoptosis, late apoptosis, necrosis and normal cells rates in ALF-derived ADMSCs and normal ADMSCs at passage 5; (**C**) Early apoptosis, late apoptosis, necrosis and normal cells rate in ALF-derived ADMSCs and normal ADMSCs at passage 10.

Flow cytometry (Figure 3A) revealed that both ALF-derived ADMSCs and normal ADMSCs at passage 5 presented low rates of cell apoptosis. All of the recorded rates are shown in detail in Figure 3B,C. The rates of early apoptosis in ALF-derived ADMSCs were 0.65% ± 0.39% at passage 5 and 4.85% ± 5.87% at passage 10. The rates of early apoptosis in normal ADMSCs were 1.13% ± 0.71% at passage 5 and 1.25% ± 0.78% at passage 10. The rates of late apoptosis in ALF-derived ADMSCs were 1.3% ± 0.51% at passage 5 and 5.55% ± 0.78% at passage 10. The rates of late apoptosis in normal ADMSCs were 2.7% ± 2.33% at passage 5 and 8.05% ± 3.18% at passage 10. The rates of necrosis in ALF-derived ADMSCs were 0.58% ± 0.53% at passage 5 and 4.75% ± 6.7% at passage 10. The rates of necrosis in normal ADMSCs were 0.97% ± 0.60% at passage 5 and 5.2% ± 4.95% at passage 10. The percentages of normal cells among ALF-derived ADMSCs were 97.53% ± 0.98% at passage 5 and 84.9% ± 0.14% at passage 10. The percentages of normal ADMSCs were 95.2% ± 3.63% at passage 5 and 85.5% ± 0.99% at passage 10. There was no difference in apoptosis between the ALF-derived ADMSCs and normal ADMSCs at passage 5 and passage 10.

### 2.4. Surface Antigen Expression

**Figure 4:**
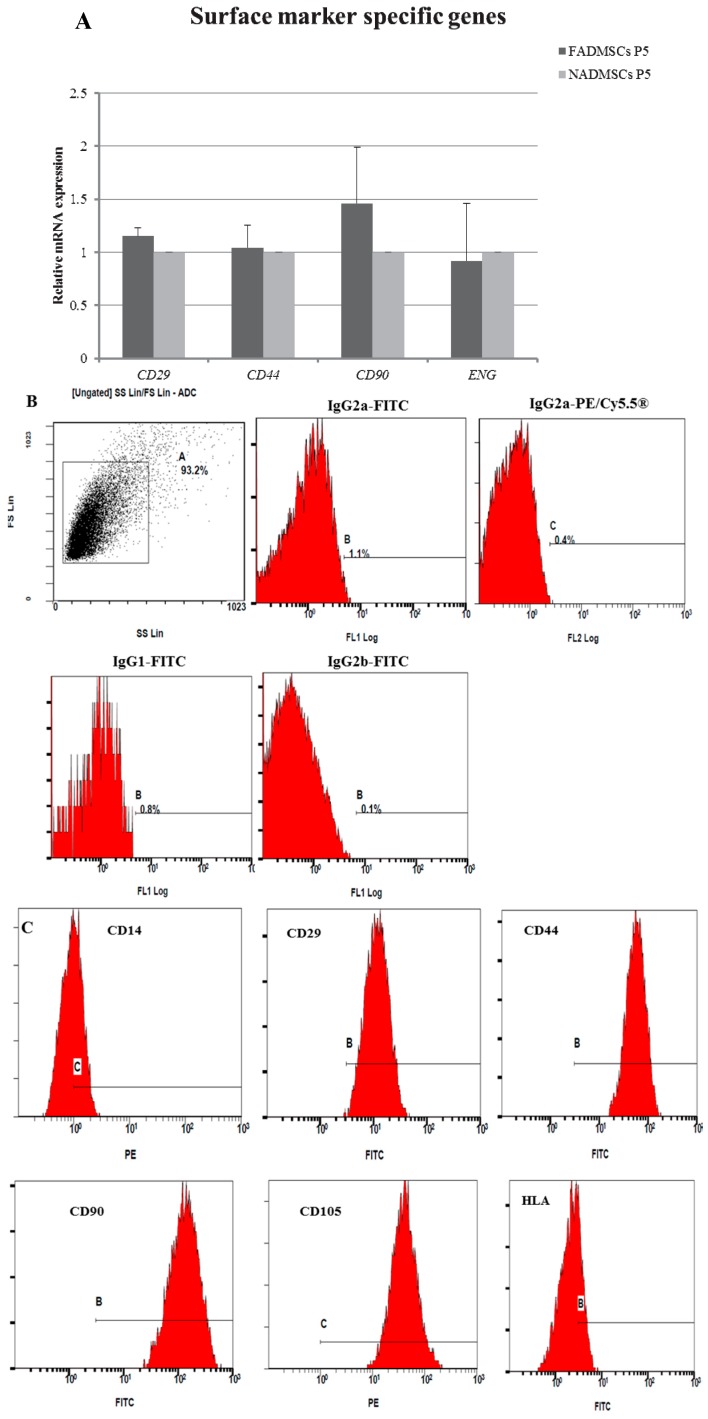

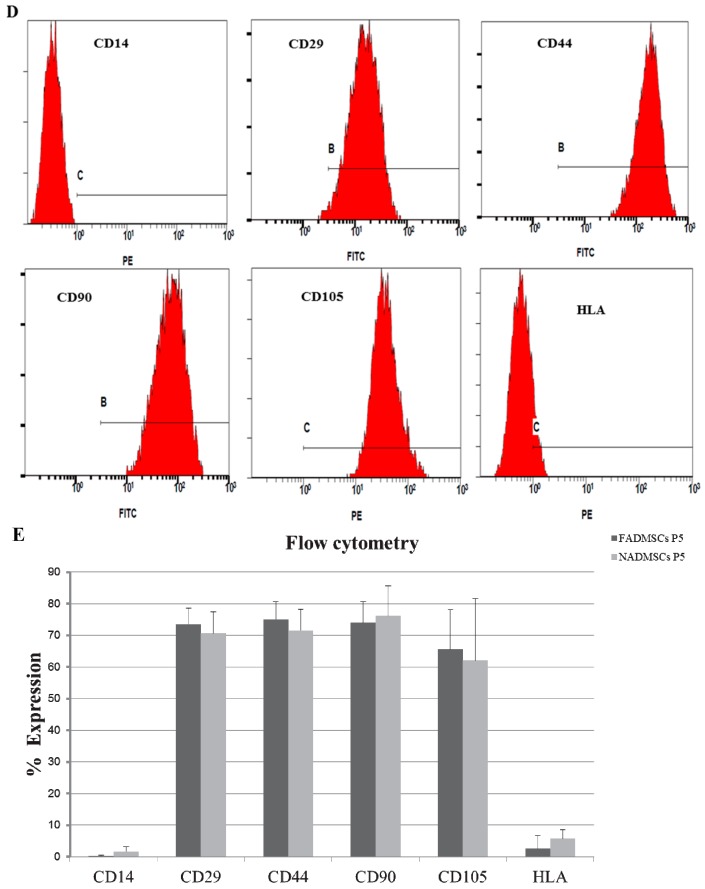
Surface antigen expression in ALF-derived ADMSCs and normal ADMSCs at the mRNA and protein levels. (**A**) Gene expression of surface antigens in ALF-derived ADMSCs and normal ADMSCs. The relative expression levels represent the level of expression normalized to GAPDH. The normal ADMSCs group was normalized to 1. Mean ± SD values are plotted; (**B**) Immunophenotype of the negative controls determined by flow cytometry using labeled antibodies; (**C**) Immunophenotype of ALF-derived ADMSCs determined by flow cytometry using labeled antibodies; (**D**) Immunophenotype of normal ADMSCs determined by flow cytometry using labeled antibodies; (**E**) Flow cytometry analysis of ALF-derived ADMSCs and normal ADMSCs revealed the negativity for hematopoietic lineage specific markers such as CD14 and HLA and positivity for mesenchymal specific antigens such as CD29, CD44, CD90 and CD105. There was no significant difference between the two groups. The results are representative of three independent experiments. Mean ± SD values are plotted.

The mRNA levels of four positive MSC-specific surface antigens including integrin β 1 (CD29), CD44 molecule (CD44), Thy-1 cell surface antigen (CD90) and endoglin (ENG) were detected in ALF-derived ADMSCs and normal ADMSCs at passage 5 (Figure 4A). There was no difference in any of these markers between the two groups detected at the mRNA level.

Then, we detected the expression levels of mesenchymal markers (CD29, CD44, CD90, and CD105) and hematopoietic and endothelial markers including CD14 and major histocompatibility complex, class II (HLA) (Figure 4B–D) of ALF-derived ADMSCs and normal ADMSCs by flow cytometry. The results demonstrated that ADMSCs isolated from ALF pigs were positive for CD29 (73.47% ± 5.10%), CD44 (75.03% ± 5.51%), CD90 (73.91% ± 6.63%) and CD105 (65.50% ± 12.53%), but negative for CD14 (0.17% ± 0.35%) and HLA (2.50% ± 4.10%) (Figure 4E). Normal ADMSCs were also positive for CD29 (70.60% ± 6.80%), CD44 (71.52% ± 6.73%), CD90 (76.07% ± 9.52%) and CD105 (62.03% ± 19.53%), but negative for CD14 (1.55% ± 1.54%) and HLA (5.67% ± 2.86%) (Figure 4E). The results demonstrated that there were no significant differences in mesenchymal surface antigens between ALF-derived ADMSCs and normal ADMSCs at the protein level.

### 2.5. Mitochondrial and Lysosomal Activities

**Figure 5:**
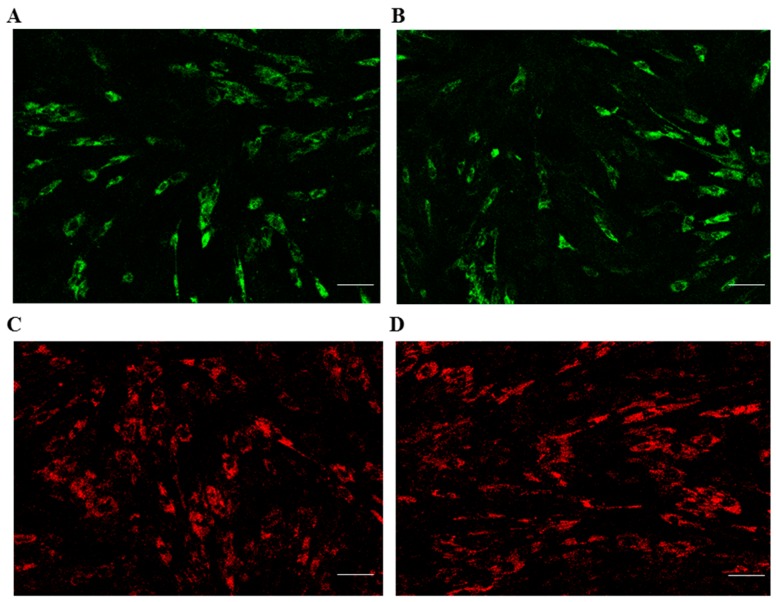
Mitochondrial and lysosomal activities of ALF-derived ADMSCs and normal ADMSCs at passage 5. (**A**) MitoTracker fluorescent staining of ALF-derived ADMSCs; (**B**) MitoTracker fluorescent staining of normal ADMSCs; (**C**) LysoTracker fluorescent staining of ALF-derived ADMSCs; (**D**) LysoTracker fluorescent staining of normal ADMSCs. Scale bars = 100 μm.

Evidence has shown that the mitochondria contribute to the maintenance of human stem cell pluripotency and culture homeostasis [15]. Furthermore, mitochondrial transfer in MSCs is able to prevent cell death caused by mitochondrial dysfunction [16]. Lysosomes are single-membrane vesicles that are found in almost all eukaryotic cells [17]. LysoTracker dye, a kind of soluble small molecule, can be used to detect programmed cell death in differentiated embryonic stem cells [18]. Mitochondrial and lysosomal activities indicate the stem cell characteristics of MSCs. The mitochondrial and lysosomal activities of both ADMSCs at passage 5 were performed by staining the cells with the MitoTracker and LysoTracker fluorescent dye (Figure 5). The results showed that the mitochondrial and lysosomal activities were similar between ALF-derived ADMSCs and normal ADMSCs.

### 2.6. Multilineage Potency

**Figure 6:**
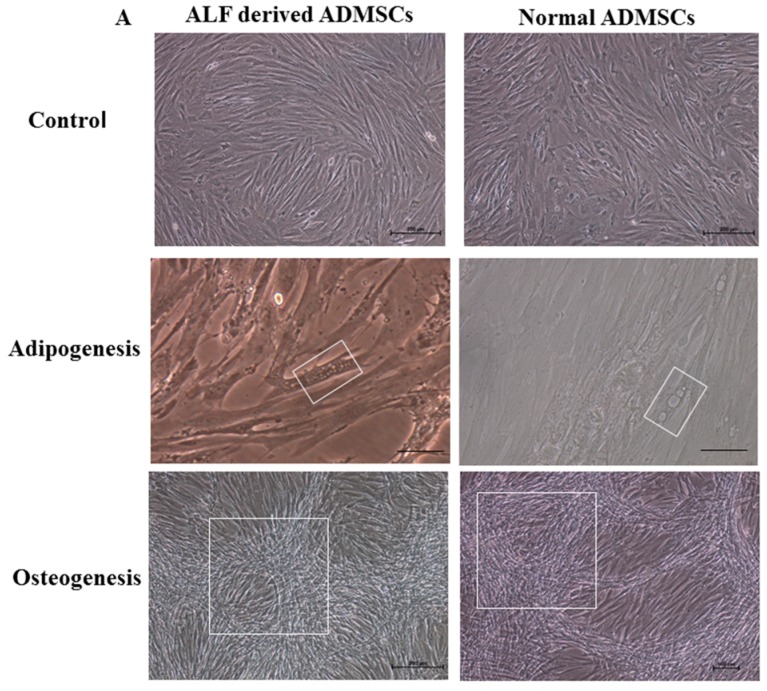

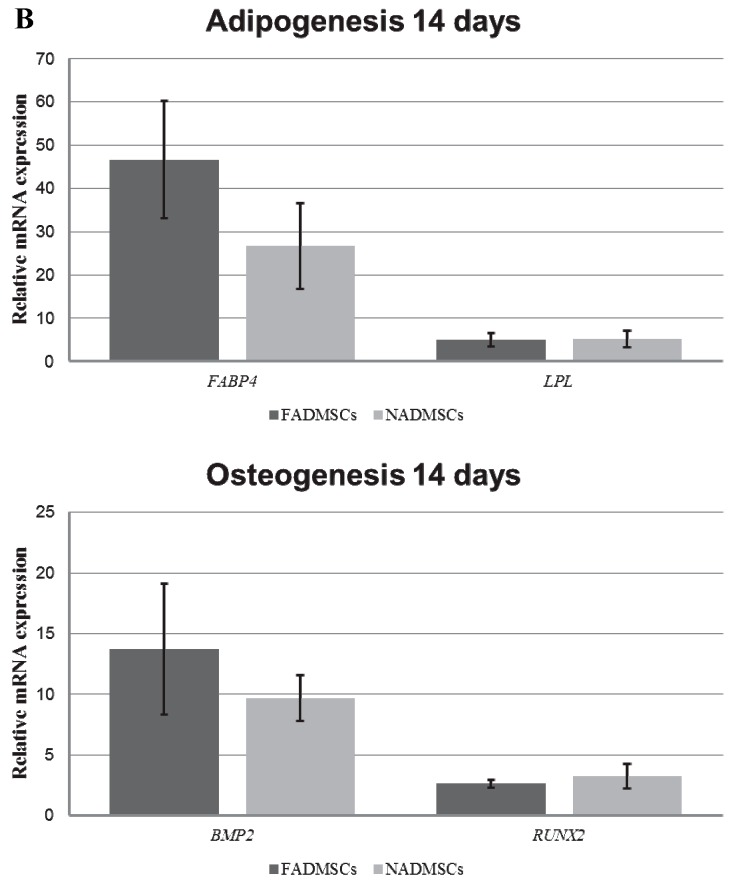
Adipogenic differentiation and osteogenic differentiation of ALF-derived ADMSCs and normal ADMSCs at passage 5. (**A**) The cell morphology transition of adipogenic and osteogenic cells after 14 days under a microscope: The white boxes of the second row indicate the lipid accumulation in adipogenic ADMSCs; The white boxes of the last row indicate the calcium accumulation in osteogenic ADMSCs. The first row and the last row: Scale bars = 200 µm; the second row: Scale bars = 50 µm; (**B**) The mRNA expression levels of adipogenic markers and osteogenic markers were significantly increased after 14 days. The relative expression levels in undifferentiated ALF-derived ADMSCs and normal ADMSCs were normalized to 1. The results are representative of three independent experiments. Mean ± SD values are plotted.

To identify the multilineage differentiation abilities of ALF-derived ADMSCs, we induced ALF-derived ADMSCs and normal ADMSCs to differentiate into adipocytes and osteocytes for 14 days (Figure 6A). The ADMSCs from both groups showed lipid accumulation after adipogenic differentiation for 14 days and calcium accumulation after osteogenic differentiation for 14 days. Then, the mRNA expression of adipogenic markers including fatty acid binding protein 4 (FABP4) and lipoprotein lipase (LPL) and osteogenic markers including bone morphogenetic protein 2 (BMP2) and runt-related transcription factor 2 (RUNX2) was detected via RT-QPCR (Figure 6B). Both of the adipogenic markers were significantly increased in ALF-derived ADMSCs and normal ADMSCs after adipogenic differentiation for 14 days, and the osteogenic markers were significantly increased in ALF-derived ADMSCs and normal ADMSCs after osteogenic differentiation for 14 days. Our results showed that the multilineage potency of ADMSCs was not altered by ALF.

### 2.7. Liver-Specific Gene Expression

**Figure 7:**
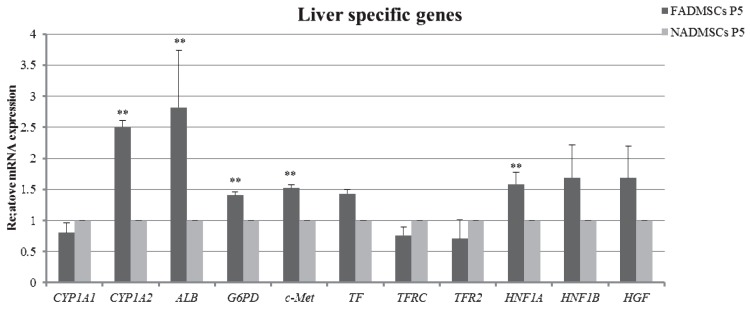
Real-Time Quantitative-PCR (RT-QPCR) analysis of liver-specific gene expression in ALF-derived ADMSCs and normal ADMSCs. The relative expression levels in normal ADMSCs were normalized to 1. Mean ± SD values are plotted. ** *p* < 0.01.

The results of RT-QPCR analysis of ALF-derived ADMSCs and normal ADMSCs to reveal the expression of liver-specific genes is demonstrated in Figure 7. Cytochrome P-450 (CYP) 1A2, albumin (ALB), glucose-6-phosphate dehydrogenase (G6PD), hepatocyte growth factor receptor (c-Met), and hepatocyte nuclear factor-1α (HNF1A) were significantly up-regulated in ALF-derived ADMSCs compared with normal ADMSCs. CYP1A1, transferrin (TF), transferrin receptor (TFRC), transferrin receptor 2 (TFR2), hepatocyte nuclear factor-1β (HNF1B) and hepatocyte growth factor (HGF) presented comparable expression levels between the two groups.

## 3. Discussion

In this study, we confirmed the ALF group and normal group by liver histopathology, then the morphological characteristics of ALF-derived ADMSCs and normal ADMSCs included a long spindle in clonogenic cells grown under culture conditions. The senility of the ADMSCs in the two groups was similar at an early age and even when cultured to higher passage numbers. The elongated shape of the cells became more cubic after passage 10, while a round or flat shape was observed at later passage, indicating senility. The proliferate ability and apoptosis rates of both cells were comparable at early or late passage. There are few specific markers for porcine MSCs, and many of the positive mesenchymal markers described for other species show little or no cross-reactivity in porcine animals [19,20]. In this work, we analyzed four surface markers through RT-QPCR and six surface markers through flow cytometry. The mRNA expression levels of four surface markers in ALF-derived ADMSCs was comparable to that in normal ADMSCs. Although the levels of positive surface antigens were not as high as those obtained in humans and rats in previous studies [21], the trend was consistent with the general standards for MSCs. As CD29, CD44, CD90 and CD105 are considered to be human mesenchymal markers, they may also be used to identify porcine MSCs [21]. The pattern of marker expression established for MSCs from other species may not always be the same as that for human MSCs [22]. Noort *et al.* [23] also demonstrated that surface antigens such as Stro-1, W4A5B5, W3C4 and CD146 were expressed on culture-expanded MSCs, but in lower percentages in porcine MSCs than in human MSCs. Our results indicated that both ALF-derived porcine ADMSCs and normal porcine ADMSCs expressed mesenchymal markers but did not express hematopoietic markers. The mitochondria are organelles that play pivotal roles in a range of diverse cellular functions, from energy generation to redox homeostasis and apoptosis regulation. Lysosomes are single-membrane vesicles that are found in almost all eukaryotic cells [17] and can be used to indicate programmed cell death in differentiated embryonic stem cells [18]. Mitochondrial and lysosomal activities were comparable between ALF-derived ADMSCs and normal ADMSCs, and mitochondrial function has been suggested as an indicator of stem cell activities [24]. The number and morphology of mitochondria are transiently associated with the pluripotent state [25,26]. A relatively underdeveloped mitochondrial network and low mitochondrial activity appear to be features of “stemness”, as confirmed *in vivo* in spermatogonia, the inner cell mass, and early embryos [27]. Taken together, our results indicated that ALF-derived ADMSCs show comparable biological activity to normal ADMSCs. To determine the multilineage potency of ALF-derived ADMSCs, we induced the cells to differentiate into adipocytes and osteocytes. The mRNA expression levels of tissue-specific markers suggested that the multilineage potency of ADMSCs was maintained after ALF. The results regarding surface antigens and multilineage differentiation may indicate that ADMSCs retain MSC characteristics after ALF.

Previous evidence has demonstrated that ALF-derived BMMSCs expressed liver-specific genes during early differentiation and possessed greater hepatogenic potency *in vitro* compared with control group [28]. Meanwhile, the majority of the genes were observed to be up-regulated in ALF-derived BMMSCs [29]. In addition, pretreatment with injured liver tissue enhanced the hepatic differentiation ability of MSCs and then improved the therapeutic efficacy of liver fibrosis [30]. When incubated with serum collected from rats after partial hepatectomy, ADMSCs changed to a round or polygonal shape with transient upregulation of hepatic functions *in vitro* [31]. Katoonizadeh *et al.* [32] demonstrated that patients with higher scores in a model for end-stage liver disease show greater levels of hepatic progenitor cell activation compared with the normal liver; moreover, patients with ALF present an increased degree of hepatic progenitor cell proliferation compared with the native liver. MSCs are responsive to adapt their functions to the environment, though it remains unclear whether they promote regeneration by differentiating into terminal cell types or producing cytokines [33,34]. In the present study, the basal expression levels of liver-specific genes in ALF-derived ADMSCs were higher than in normal ADMSCs, which may be attributed to the effect of the injured *in vivo* environment. Based on our findings, we suggest that ALF-derived ADMSCs may serve as a candidate source for clinical usage in end-stage liver diseases. Bruckner *et al.* [35] showed that porcine ADMSCs were able to serve as a functional liver support after surgical intervention in the pig model. In addition, Zhou *et al.* [36] demonstrated that MSCs was as efficient as the hepatocyte like cells, and they could stimulate the host hepatocyte regeneration in ALF.

In conclusion, ALF does not influence the morphology, proliferative ability, apoptosis rates, expression of MSC-specific markers, mitochondrial and lysosomal activities, and multilineage potency of ADMSCs. In addition, ADMSCs derived from ALF pigs were found to express more liver-specific genes; therefore, our work was motivated by the hypothesis that ADMSCs likely express more genes involved in hepatogenesis prior to migration to the liver and differentiation into hepatocyte-like cells during severe hepatic damage. Adipose tissue presents the benefits of abundance, easier extraction and reduced injury to patients. Hence, we suggest that ALF-derived ADMSCs may serve as a resource for hepatocyte and liver regeneration, and our findings further confirm their potential as a stem cell-based therapy for end-stage liver diseases. MSCs hold great promise for clinical translation, though assessment of the safety and feasibility of their use in a large animal model will be necessary before their first applications in humans. Porcine MSCs are particularly suitable for the generation of adult stem cell-based liver cell therapeutics to perform proof-of-concept and safety analyses in a pre-clinical setting and in terms of regulatory issues, in this highly relevant large animal model.

## 4. Experimental Section

### 4.1. Animals

Six male miniature Bama pigs (Pig Breeding Center of Taihe Biotechnology, Jiangsu, China) weighing 18–23 kg were used, with approval of the Animal Care Ethics Committee of Zhejiang Academy of Traditional Chinese Medicine (approval no: ZJATCM 20140017; 1 April 2014). Each pig was housed in a single standard cage in an air-conditioned room (20–25 °C), with a 12-h light and 12-h dark cycle. A standard laboratory diet and water were provided. The pigs were allowed to acclimate to the experimental environment for a minimum of one week before being used in this study. The experimental room and all associated facilities remained unchanged throughout the experimental period.

### 4.2. d-Galactosamine-Induced ALF Model and Immunohistochemistry

All experimental protocols and study methods were approved by the Animal Care Ethics Committee of the First Affiliated Hospital, School of Medicine, Zhejiang University. ALF was induced with d-galactosamine, as described previously by our group [37]. Animals were fasted for 8 h prior to catheterization. Prior to catheterization, Ketamine (4 mg/kg; Fujian Gutian Pharmaceutical Co., Ltd., Fujian, China) was injected to induce sedation intramuscularly through the external jugular vein. Continuous anesthesia was provided through auricular vein administration of propofol (AstraZeneca, Caponago, Italy) at 2.5 mg/kg/h during the operation. The right jugular vein was cannulated with a 6.5-F dual-lumen catheter (Baihe Biotechnology, Guangdong, China) for blood sampling, drug administration, and treatment. Catheterization and anesthesia were completed within 30 min. Then, the pigs were returned to their cages after awakening from anesthesia. d-galactosamine (Hanhong Chemical Co., Ltd., Beijing, China) dissolved in 5% glucose (pH 6.8) was infused through the external jugular venous catheter without anesthesia 36 h after catheterization. Sex, age, and weight-matched sham-operated animals (which underwent a similar procedure but without ALF induction) were also included in the experiment. Both groups contained three pigs, as determined by a random drawing. Liver tissues were collected for histochemistry and immunohistochemistry. After the experimental pigs died, liver tissues were collected within 30 min, fixed by 10% formalin for 24 h, and then treated with paraffin imbedding. All specimens were analysed by hematoxylin and eosin (HE) staining. The analysis was performed by a procedure described previously [38].

### 4.3. Isolation, in Vitro Culture, and Morphology of ADMSCs

After d-galactosamine induced for 60 h, sub-peritoneal adipose tissues were extracted from ALF and normal pigs under aseptic condition. The capillaries were removed and the adipose tissue was washed several times with phosphate-buffered solution (PBS, pH 7.2 ± 0.1, GenomSciences, Hangzhou, China) under rigorous aseptic conditions. The tissue was then minced prior to digestion with a 0.1% collagenase solution (Gibco, Carlsbad, CA, USA) for 90–120 min at 37 °C. Then the mixture was centrifuged (1000 rpm, 10 min) at room temperature (25 °C), and the supernatant was discarded. Then the collected cells were washed with PBS (1000 rpm, 5 min) for several times, they were re-suspended in Low glucose-Dulbecco’s Modified Eagle Medium (L-DMEM) (Gibco, Carlsbad, CA, USA) containing 10% fetal bovine serum (FBS) (Gibco, Carlsbad, CA, USA) and 1% penicillin and streptomycin (Gibco, Carlsbad, CA, USA). Cells were subsequently seeded into culture flasks (T25; Corning Inc., Corning, NY, USA) and maintained in a humidified atmosphere of 95% air and 5% CO_2_ at 37 °C. When the cells reached 80%–90% confluence, they were collected with 0.25% Trypsin-EDTA (Gibco, Carlsbad, CA, USA) and diluted 1:2 or 1:3 at each passage. The morphological properties of the cultured ADMSCs were observed with an inverted phase-contrast microscope (Nikon, ECLIPAS TS100, Tokyo, Japan). Observations were performed regularly and images were acquired with digital imaging software (NIS-Elements F3.0, Tokyo, Japan).

### 4.4. Analysis of Cell Proliferation

Fifth, tenth and fifteenth ADMSCs from both groups were plated into 96-well plates (Nunclon™Δ Surface, Roskilde, Denmark) at a density of 2000 cells per well. Over the following 7 days, 10 µL of the WST-1 Cell Proliferation Reagent (5 mg/mL, Roche, Basel, Switzerland) was added to every six wells, followed by incubation for 1 h in a CO_2_ incubator once daily. Replacement of the culture medium was performed every two days. The absorbance at 450 nm was measured using a spectrophotometer (Beckman Coulter^®^ Multimode Detector DTX880, Beckman Coulter, Inc.; Los Angeles, CA, USA), OD values were plotted as a function of elapsed time. The experiments were repeated at least three times.

### 4.5. Detection of Cellular Apoptosis

Cellular apoptosis was performed by flow cytometry with the Annexin V/PI apoptosis kit (BD Bioscience Pharmingen Inc., San Diego, CA, USA). ADMSCs were rinsed twice or three times with PBS and re-suspended in binding buffer (1×), and the cell concentration was adjusted to 1 × 10^6^ cells/mL. 100 µL of the cell suspension was transferred into the flow cytometry tube, after that, 5 µL of Annexin V/FITC and 10 µL of propidium iodide were added. Then the cell suspension was mixed adequately and then incubated at room temperature for 15 min in the dark. Next, 400 µL of PBS was added into the tube, and the apoptosis rates were analyzed with a Beckman Coulter Cytomics FC 500 MPL (Beckman Coulter, Inc., Los Angeles, CA, USA). The experiments were repeated three times.

### 4.6. Flow Cytometry Analysis

The fifth-passage ALF-derived ADMSCs and ADMSCs from control healthy pigs were washed with PBS-containing 0.3% (*w*/*v*) FBS, and the concentration was adjusted to 1 × 10^6^ cells/100 μL. The cells were examined for negative controls, MSC markers and hematopoietic markers through incubation with antibodies including a mouse monoclonal antibody for CD14-phycoerythrin (PE) (10 µL/10^6^ cells; Abcam, Cambridge, UK), a mouse monoclonal antibody for CD29-fluorescein isothiocyanate (FITC) (1.5 µL/10^6^ cells; Abcam, UK), a rat monoclonal antibody for CD44-FITC (1:50; Abcam, UK), a mouse monoclonal antibody for CD90-FITC (4 µL/10^6^ cells; Abcam, UK), a mouse monoclonal antibody for CD105-PE (20 µL/10^6^ cells; Abcam, UK) and a mouse monoclonal antibody for HLA DR + HLA DP-FITC (20 µL/10^6^ cells; Abcam, UK). Antibodies including mouse IgG2a-FITC (1:50; Abcam, UK), mouse IgG2a-PE/Cy5.5^®^ (10 µL/10^6^ cells; Abcam, UK), mouse IgG1-FITC (10 µL/10^6^ cells; Abcam, UK) and rat IgG2b-FITC (10 µL/10^6^ cells; Abcam, UK) were used as isotype controls. After incubated with antibodies at room temperature for 30 min in the dark, the cells were washed several times with PBS. Flow cytometry analysis was performed with a Beckman Coulter Cytomics FC 500 MPL (Beckman Coulter, Inc.; Los Angeles, CA, USA).

### 4.7. Measurement of Mitochondrial and Lysosomal Fluorescence

Mito-Tracker Green FM (Invitrogen Life Technologies, Carlsbad, CA, USA) and Lyso-Tracker Red (Invitrogen Life Technologies) were diluted in DMEM to the appropriate concentrations. The fifth-passage ADMSCs were incubated with pre-warmed (37 °C) probe-containing culture medium for Lyso-Tracker Red and Mito-Tracker Green FM according to the protocols. The fluorescence of the cells was observed and photographed under a Fluorescent microscope (ZEISS LSM710, BioSciences, Jena, Germany).

### 4.8. Adipogenic Differentiation and Osteogenic Differentiation

To induce adipogenesis, ALF-derived ADMSCs and normal ADMSCs from passage 5 at a density of 1 × 10^5^/well in 6-well plates were treated with adipogenic medium (OriCell™ hMSC Adipogenic Differentiation Medium, Cyagen Biosciences, Guangzhou, China) as per the protocol. The medium was changed every three days. After 2 weeks, mRNA was extracted from the two groups. For osteogenic differentiation, ALF-derived ADMSCs and normal ADMSCs from passage 5 at a density of 5 × 10^4^/well in 6-well plates were treated with osteogenic medium (OriCell™ hMSC Osteogenic Differentiation Medium, Cyagen Biosciences, Guangzhou, China) according to the protocol. The medium was changed every three days. After 2 weeks, mRNA was extracted from the two groups.

### 4.9. RNA Extraction, Reverse Transcription (RT) and Real-Time Quantitative-PCR (RT-QPCR)

Using the RNAiso plus kit (TaKaRa, Tokyo, Japan), the total RNA of fifth ADMSCs was isolated according to the manufacturer’s protocol. The RNA was first treated with DNase (TaKaRa, Tokyo, Japan) in a 10 μL reaction with 5× gDNA Easer Buffer (2 μL), gDNA Easer (1 μL) and total RNA (1 μg). The reaction was conducted at 42 °C for 2 min. For the mRNAs, the PrimeScript RT reagent Kit (TaKaRa, Tokyo, Japan) was used for RT in a total volume of 20 μL, with 4 μL of 5× PrimeScript Buffer PCR buffer, 1 μL of PrimeScript RT enzyme mix I, 1 μL of RT Primer Mix, and 10 μL of the RNA sample. The RT reaction was initiated with a 15 min incubation period at 37 °C and ended after a 5 s enzyme denaturing step at 85 °C. Real-time amplification was performed with SYBR Premix Ex Taq (TaKaRa, Tokyo, Japan) in an ABI 7900 thermocycler (ABI; Foster City, CA, USA). RT-QPCR was carried out in a 10 μL reaction system, containing 5 μL of SYBR Premix Ex Taq (2×), 0.4 μL of PCR primers (10 μM), 0.2 μL of ROX reference dye, and 1 μL of cDNA. The PCR thermal cycling parameters were 95 °C for 30 s, followed by 40 cycles of 95 °C for 5 s and 60 °C for 30 s. After RT-QPCR, a dissociation curve was constructed at 95 °C for 15 s, 60 °C for 15 s, and 95 °C for 15 s for the detection of PCR product specificity. Each reaction was performed in triplicate. The reference gene GAPDH was used as a relative control for expression levels. The relative alteration was calculated using the cycle threshold (*C*t) method. The calculation of gene expression was performed as follows using comparative cerebral CD29 expression from the IVF group as an example: CD29 = 2 − ΔΔ*C*t; ΔΔ*C*t = (*C*t_CD29_ − *C*t_GAPDH_) ALF − (*C*t_CD29_ − *C*t_GAPDH_ _control_). The expression of genes encoding the cell surface antigens CD29, CD44, CD90 and ENG was examined. The evaluated adipogenic genes were FABP4 and LPL. The assessed osteogenic genes were BMP2 and RUNX2. The evaluated liver-specific genes were CYP1A1, CYP1A2, ALB, G6PD, c-Met, TF, TFRC, TFR2, HNF1A, HNF1B and HGF. The primers (Sangon, Shanghai, China) used in these assays are shown in Table 1.

**Table 1:** Primers for RT-QPCR.

Gene Name	NCBI Accession No.	Product Size (bp)	Forward	Reverse
*CD29*	NM_213968.1	131	5′-CACTGCTGCTCATTTGGAAG-3′	5′-GGTTGTCACGGCACTCTTAT-3′
*CD44*	XM_003122867.4	109	5′-TGGAAGAGAGAAAGCCAAGC-3′	5′-GCCGTCATAAACTGGTCTGG-3′
*CD90*	NM_001146129.1	125	5′-GGCATCGCTCTCTTGCTAAC-3′	5′-GGCAGGTTGGTGGTATTCTC-3′
*ENG*	NM_214031.1|	121	5′-AGGTTTCTGAGGGCTGTGTG-3′	5′-TTTGGGTTGGTCATCTGGAC-3′
*CYP3A4*	NM_214423.1	220	5′-TTACACTTACCTGCCCTTTGG-3′	5′-TCCACTTACGGTCCCATCTC-3′
*ALB*	NM_001005208.1	241	5′-GCCTCTTGTGGATGAGCCTA-3′	5′-GTTCAGGACCAGGGACAGAT-3′
*G6PD*	XM_003360515.3	104	5′-GATCTACCGCATCGACCACT-3′	5′-TGTTGTCTCGGTTCCAGATG-3′
*c-MET*	NM_001038008.1	113	5′-GTGGCTGGGACTTTGGATT-3′	5′-CGTGTTTGTCGTGCTCTCAC-3′
*TF*	NM_001244653.1	101	5′-GAGGCCAATAAGTGCTCCAG-3′	5′-ATGCAATCCAGGTAGGAGGA-3′
*TFRC*	NM_214001.1	111	5′-TCCCTCAAACACCTCGCTTA-3′	5′-GGGCATAATCTTCATTCAGCA-3′
*TFR2*	XM_003124374.3	108	5′-TGATAAGTTCCACGCCAAGA-3′	5′-ACCTGCTCGTAAAGGGTCTG-3′
*HNF1A*	NM_001032388.1	103	5′-AGGCTCGTGATTCTGCACTT-3′	5′-TTTGGCCTTACTGCCTTCTG-3′
*HNF1B*	NM_213956.1	106	5′-TGTCTACCTTGTGCTCCTTCG-3′	5′-CAGTGTGTTTGGCTCAGTTCA-3′
*HGF*	XM_003130222.3	104	5′-ATGCGAGGGAGATTATGGTG-3′	5′-GACGATTTGGAATGGCACA-3′
*GAPDH*	NM_001206359.1	154	5′-ATGGTGAAGGTCGGAGTGAA-3′	5′-CGTGGGTGGAATCATACTGG-3′

### 4.10. Statistical Analysis

The data are presented as the mean ± SD. The data were assessed by Student’s *t*-test with SPSS software (version 20.0; SPSS Inc., Chicago, IL, USA). Differences were considered significant at *p* < 0.05.

## Abbreviations

MSCs, mesenchymal stem cells; BMMSCs, bone marrow mesenchymal stem cells; ADMSCs, adipose-derived mesenchymal stem cells; ALF, acute liver failure; HGF, hepatocyte growth factor; AFP, α fetal protein; RT-QPCR, real-time quantitative PCR; FITC, fluorescein isothiocyanate; PE, phycoerythrin; CD29, integrin β 1; CD44, CD44 molecule; CD90, Thy-1 cell surface antigen; ENG, endoglin; FABP4, fatty acid binding protein 4; LPL, lipoprotein lipase; BMP2, bone morphogenetic protein 2; RUNX2, runt-related transcription factor 2; CYP, cytochrome P-450; ALB, albumin; G6PD, glucose-6-phosphate dehydrogenase; c-Met, hepatocyte growth factor receptor; TF, transferrin; TFRC, transferrin receptor; TFR2, transferrin receptor 2; HNF1A, hepatocyte nuclear factor-1α; HNF1B, hepatocyte nuclear factor-1β.
